# Targeting microglial NLRP3 in the SNc region as a promising disease‐modifying therapy for Parkinson's disease

**DOI:** 10.1002/brb3.2784

**Published:** 2022-10-05

**Authors:** Chen Qiao, Tao Dang, Yan Zhou, Yuan‐Zhang Zhou, Rong Zhao, Min Wang

**Affiliations:** ^1^ Department of Clinical Pharmacy The Affiliated Hospital of Jiangsu University, Jiangsu University Zhenjiang Jiangsu China; ^2^ College of Pharmacy Jiangsu University Zhenjiang Jiangsu China; ^3^ Department of Clinical Pharmacy Shanghai General Hospital, Shanghai Jiao Tong University School of Medicine Shanghai China; ^4^ Department of Pharmacology Nanjing Medical University Nanjing Jiangsu China; ^5^ Department of Geriatrics Affiliated Brain Hospital of Nanjing Medical University Nanjing Jiangsu China

**Keywords:** disease‐modifying therapy, dopaminergic neuron, microglia, NLRP3, Parkinson's disease

## Abstract

**Introduction:**

Parkinson's disease (PD) is a neurodegenerative disorder characterized by progressive loss of dopaminergic (DA) neurons. Accumulating evidence has shown that activation of the NLR family pyrin domain‐containing 3 (NLRP3) inflammasome is an early and cardinal feature in PD progression. Nevertheless, little is known about the effect of NLRP3 in the substantia nigra pars compacta (SNc) on DA neurodegeneration.

**Methods and results:**

In the present study, we constructed NLRP3 interference sequences wrapped by lentivirus (LV3‐siNlrp3) to facilitate NLRP3 knockdown in the SNc region by intracerebral stereotactic injection. Then, we explored the effects of NLPR3 knockdown on PD pathologies via behavioral monitoring, immunohistochemistry and western blot analysis in acute 1‐methyl‐4‐phenyl‐1, 2, 3, 6‐tetrahydropyridine (MPTP) mouse model. Moreover, we performed in vitro experiments to investigate the effect of microglial NLRP3 knockdown on DA neuron survival in the context of 1‐methyl‐4‐phenylpyridinium (MPP^+^) stimulation. Our results demonstrated that NLRP3 knockdown in the SNc region significantly improved MPTP‐induced dyskinesia, DA neuronal loss and microglia activation in vivo. Meanwhile, knockdown of microglial NLRP3 attenuated MPP^+^‐induced DA neuronal damage in an indirect coculture system in which neurons were cultured in microglial conditional medium. Cumulatively, these data reveal that microglial NLRP3 located in the SNc region is detrimental to DA neurons survival, and knockdown of microglial NLRP3 is a potential strategy to rescue DA neurons in the progression of PD.

**Conclusions:**

This work demonstrates the role of NLRP3 in PD pathogenesis via microglia‐neuron communication, and sheds light on targeting microglial NLRP3 to develop disease‐modifying therapy for PD.

## Graphical Abstract

1. NLRP3 inflammasome is a crucial regulator of neuroinflammation in microglia of PD model. 2. Knockdown of NLRP3 in the SNc region ameliorates behavioral disorders and exerts protective effects on DA neurons in PD model mice. 3. Targeting microglial NLPR3 provides a promising disease‐modifying target for PD therapy.

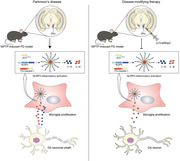

## 1 INTRODUCTION

Parkinson's disease (PD) is the second major neurodegenerative disease in the world characterized by progressive loss of dopaminergic (DA) neurons in the substantia nigra pars compacta (SNc) (Bloem et al., 2021; Xu et al., 2012). Recent study has shown that PD has gradually become a major social problem worsening the quality of life (Li et al., 2019; Olatunji et al., 2016). To date, no clinical treatments have been developed to restrain the degeneration of DA neurons. Therefore, it is urgently needed to explore potential biomolecular targets for the development of PD therapy.

Previous studies have shown that neuroinflammation occurs earlier than DA neuronal degeneration in the progression of PD (De Virgilio et al., 2016; Hirsch & Standaert, 2021). In the brain, microglia acts as macrophage‐like cells to maintain immune defense. However, overactivation of microglia releases potent proinflammatory cytokines, such as interleukin (IL)−1β and tumor necrosis factor (TNF)‐α, which aggravate neurodegeneration and induce neuronal injury (Badanjak et al., 2021; Ho, 2019). Conversely, neuronal damage also promotes the secretion of proinflammatory cytokines, amplifying the neuroinflammation and microgliosis, thus forming a positive feedback loop (Liu et al., 2022). For this reason, neuroinflammation may be a promising target for delaying DA neuronal loss in PD.

Numerous studies have demonstrated that inflammasome activation‐mediated neuroinflammatory response is of considerable importance in the pathological process of PD (Holbrook et al., 2021). More importantly, studies by our group and other researchers have reported that NLR family pyrin domain‐containing 3 (NLRP3) inflammasome may be one of the most vital inflammasome linking central and peripheral inflammation, which is thought to be highly correlated with the development of PD (Haque et al., 2020; Lee et al., 2019). Since extensive NLRP3 inflammasome was found in the SNc of PD patients (Gordon et al., 2018), plentiful studies have focused on exploring the pathophysiological mechanism within NLRP3 and developing treatments targeting NLRP3 inflammasome activation in PD (Qiao et al., 2018; Wang et al., 2020). However, there is a lack of direct evidence showing the influence of microglial NLRP3 located in the SNc region on DA neurodegeneration.

Therefore, our study aimed to discover whether the acquired downregulation of NLRP3 in the SNc affects the development of PD. We demonstrated that knockdown of NLPR3 by injection of lentivirus‐coated NLRP3 interference sequence into the SNc could improve 1‐methyl‐4‐phenyl‐1, 2, 3, 6‐tetrahydropyridine (MPTP)‐induced PD‐like symptoms. Furthermore, we found that the acquired inhibition of NLRP3 expression in the SNc significantly impeded MPTP‐induced microglial activation, even alleviated 1‐methyl‐4‐phenylpyridinium (MPP^+^)‐induced DA neuronal damage. These findings fill the gap in our understanding of microglia‐neuron communication in the SNc, and provide a promising disease‐modifying strategy by targeting microglial NLRP3 to treat PD.

## 2 MATERIALS AND METHODS

### 2.1 Animals

All experimental animals were approved by the Institutional Animal Care and Use Committee of Jiangsu University (license No. SCXK (Su) 2018‐0053). Male C57BL/6J mice aged 3–4 months and weighing 25–30 g were obtained from the Laboratory Animal Center of Nanjing Medical University. The mice were free drinking and feed in a room temperature kept at 24°C ± 2°C and a 12 h light/dark cycle.

### 2.2 Animal model preparation

#### 2.2.1 Acute MPTP model

Mice were intraperitoneally injected with MPTP (20 mg/kg; Sigma, M0896, USA) every 2 h for 1 day (four times in all) to simulate the pathological process of PD. The mice were sacrificed at 7 days after the last administration. Mice in control group were treated with sterile saline only.

#### 2.2.2 Intracerebral stereotactic injection

Mice were fasted for 4 h prior to surgery and anesthetized by inhalation of ether. The mice were immobilized on a stereoscopic brain locator (Stoelting, Wood Dale, USA) to determine the location of the anterior fontanelle. The stereographic coordinates of the left and right SNc positions, in millimeters, are as follows: AP: ‐ 0.5 mm; ML: ± 1.3 mm; DV: ‐ 4.2 mm, and 4 μl of GFP‐labeled lentivirus mus Nlrp3 (LV3‐siNlrp3; GenePharma, Shanghai, China) were then injected into the SNc region using a Hamilton syringe at the rate of 0.25 μl/min. The control group was injected with equal amount of Negative control (NC; GenePharma, Shanghai, China). After the surgery, the mice were placed in a constant temperature and pressure environment with light and darkness for 12 h each day. After restoring for 1 week, the acute MPTP model could be prepared. The diagram of the design for animal experiments was shown in Figure 3a. The sequence of LV3‐siRNA is as follows (5′ ‐ 3′):

NC: TTCTCCGAACGTGTCACGT; LV3‐siNlrp3‐2515: GCACCCAGGCTGTAACATTCA; LV3‐siNlrp3‐2762: GGTTCTGAGCTCCAACCATTC.

### 2.3 Behavioral analysis

Seven days after last MPTP intoxication, behavioral tests prepared for all mice as described previously (Qiao et al., 2017). Rotarod test can be used to evaluate the coordination ability of limbs in mice. Three days before rotarod test, mice were trained every day to exercise at 5, 10, and 15 rpm of 5 min for each speed. Then the latency time was recorded at 15 rpm during the formal test, for a maximum of 300 s each test. The pole test is used to detect the autonomous behavior of mice. The mice was placed head upward on the top of a vertical wooden pole (diameter 1 cm, height 50 cm) and recorded two times, the time of the completely head downward (T‐Turn) and the total time to the bottom of the rod (T‐TLA). Three days before the formal experiment, the training was taken every 1 min in twice a day. The shortest time was recorded for each mouse during three trials. If the mice failed to turn completely downward, fell or slipped, which was recorded as 120 s.

### 2.4 Cell culture, transfection, and treatment of BV2 cell line

The BV2 cell is a murine microglia cell line, cells were cultured in Dulbecco's modified Eagle's medium (DMEM, Gibco, USA) containing 10% fetal bovine serum (FBS, Gibco, USA) with penicillin 100 units/ml and streptomycin 100 ug/ml, placed in a 37°C, 5% CO_2_ incubator. BV2 cells were cultured at a confluency of 70%−80% in 6‐well (or 12‐well) dishes and transfected with different lentivirus titers (10^6^ TU/ml, 0.5 × 10^7^ TU/ml, 10^7^ TU/ml) to downregulate the expression of NLRP3. After transfection with 24 h, BV2 cells were stimulated with MPP^+^ (50 μM) for 48 h, then collected cells for the following experiments.

### 2.5 Cell culture for primary mesencephalic neurons

Pregnant mice at 13–16 days were sacrificed for mesencephalic primary neurons culture. Fetal mice were washed with sterile PBS containing penicillin and streptomycin for three times, and placed in high‐glucose DMEM. The midbrain was isolated under sterile conditions with a microscope, the meninges and blood vessels were gingerly removed, 0.25% trypsin was added and digested in 37°C water bath for 8 min. Added DMEM medium containing 10% FBS and 10% horse serum, cells was shaken into single‐cell suspension, which was filtered by a 200‐mesh filter. After 24 h, the medium was half‐replaced with Neurobasal (Gibco, USA) containing 2% B27 (Gibco, USA) and 25 μM glutamine (Sigma, USA), then added with 1 μM cytarabine (Sigma, USA) after 24 h. In the next 24 h, the whole medium was replaced with Neurobasal containing 2% B27 and 25 μM glutamine. After three days of culture, the purity of neurons was higher than 95% by MAP2 (neuron maker) staining, then collected cells for the following experiments.

### 2.6 Identification and quantification of TH immunoreactivity neurons

The TH immunoreactivity of mesencephalic neurons were performed according to our previously described protocol (Qiao et al., 2016). Briefly, after incubation with the supernatant of BV2 for 6 h (Conditioned medium, CM), neurons were followed by TH immunohistochemistry. The number of TH immunoreactivity (TH‐ir) neurons was counted in 10 randomly selected fields on a Nikon inverted microscope. The values were normalized to control group. The average number of TH‐ir cells in control groups ranged from 20 to 30 per field. Each TH‐ir cell process was measured from soma to the end of the process, realized by the measurement function of Image Pro Plus 7.0.

### 2.7 Immunohistochemistry and immunofluorescence staining

The brain tissue was sequentially sectioned by Lecia frozen slicer (30 μM), and the midbrain parts were selected. For later use, immerse brain slice in 0.01 M PBS and glycerin (volume ratio 1:1) and freeze at −20°C. Slices were washed in 0.01 M PBS for three times and 3% H_2_O_2_ was added for 30 min to remove endogenous peroxidase. After rinsing with PBS again for 3× 10 min, 5% bovine serum albumin (prepared with 0.01 M PBS‐0.3% Triton X‐100) was incubated for 1 h at room temperature, then primary antibody was used, respectively: mouse anti‐TH antibody (T8700, Sigma, 1:800), rabbit anti‐Caspase‐1 (AB1871, Millipore, 1:500) and mouse anti‐Iba‐1 antibody (019‐19741, Wako, 1:1000). Afterward, appropriate secondary antibodies were used for 1 h. Incubation in DAB visualized the immunoreactivity in immunohistochemistry. Staining without primary antibodies served as a control. DAPI was used for nuclear staining in immunofluorescence.

All brain slices were 30 μm while sectioning (from approximately −2.5 mm to −3.88 mm from bregma according to Paxinos and Franklin), and one brain slice was taken at every interval of 6 for staining. To visualize, photograph, and count positive cells, specimens were observed and calculated using MicroBrightField Stereo Investigator software (MicroBrightField, USA), the actual thickness of the stained brain slice and the stereoscopic staining area were set in the system, and the number of positive cells was automatically calculated after the positive cells were marked.

### 2.8 Western blot assay

After the mouse model was successfully prepared, midbrain was isolated and weighed, 1:10 (mass: volume ratio, 10 μl/1 mg of tissue) RIPA (Beyotime, China) protein lysis solution was added and lysed on ice for 40 min. After centrifugation at 16,000 × *g* for 15 min at 4°C, the supernatant contained whole cell protein. The protein supernatant was denatured in 95°C metal bath for 5 min by adding 5× loading buffer according to volume ratio and stored at −20°C. An equivalent amount of protein (60 μg) was electrophoresed on a polyvinylidene difluoride (PVDF) membrane using sodium dodecyl sulfate and polyacrylamide gels. The PVDF membrane was placed in 10% defatted milk powder‐TBST (pH 7.4, 10 mM TriS‐HCl, 150 mM NaCl, 0.1% Tween‐20), shaken, and sealed at room temperature for 1 h, then the primary antibody prepared by 5% BSA‐TBST was added, mouse anti‐NLRP3 (AG‐20B‐0014‐C100, Adipogen, 1:1000), Rabbit anti‐Caspase‐1 (AB1871, Millipore, 1:1000), and mouse anti‐β‐actin (AC026, ABclonal, 1:1000) overnight at 4°C. PVDF was washed for 10 min × 3 times, and ECL (Pierce) was added to develop the colors by Image Quant LAS 4000 Mini (GE). The gray value of target protein was compared to internal reference β‐actin in semiquantitative analysis (Image J).

### 2.9 Statistical analysis

Results were analyzed using GraphPad Prism 8.0. Data were first examined for equal variance and then subjected to two‐way ANOVA with treatment and genotype as variables, with Tukey's post hoc tests. Student's *t*‐tests were used for single variant analyses. In all studies, *n* indicates the number of samples per group, and a critical value of *p* < .05 is used. Data are shown as means ± SEM.

## 3 RESULTS

### 3.1 Activation of the NLRP3 inflammasome exists in the SNc region of MPTP‐treated mice

**FIGURE 1:**
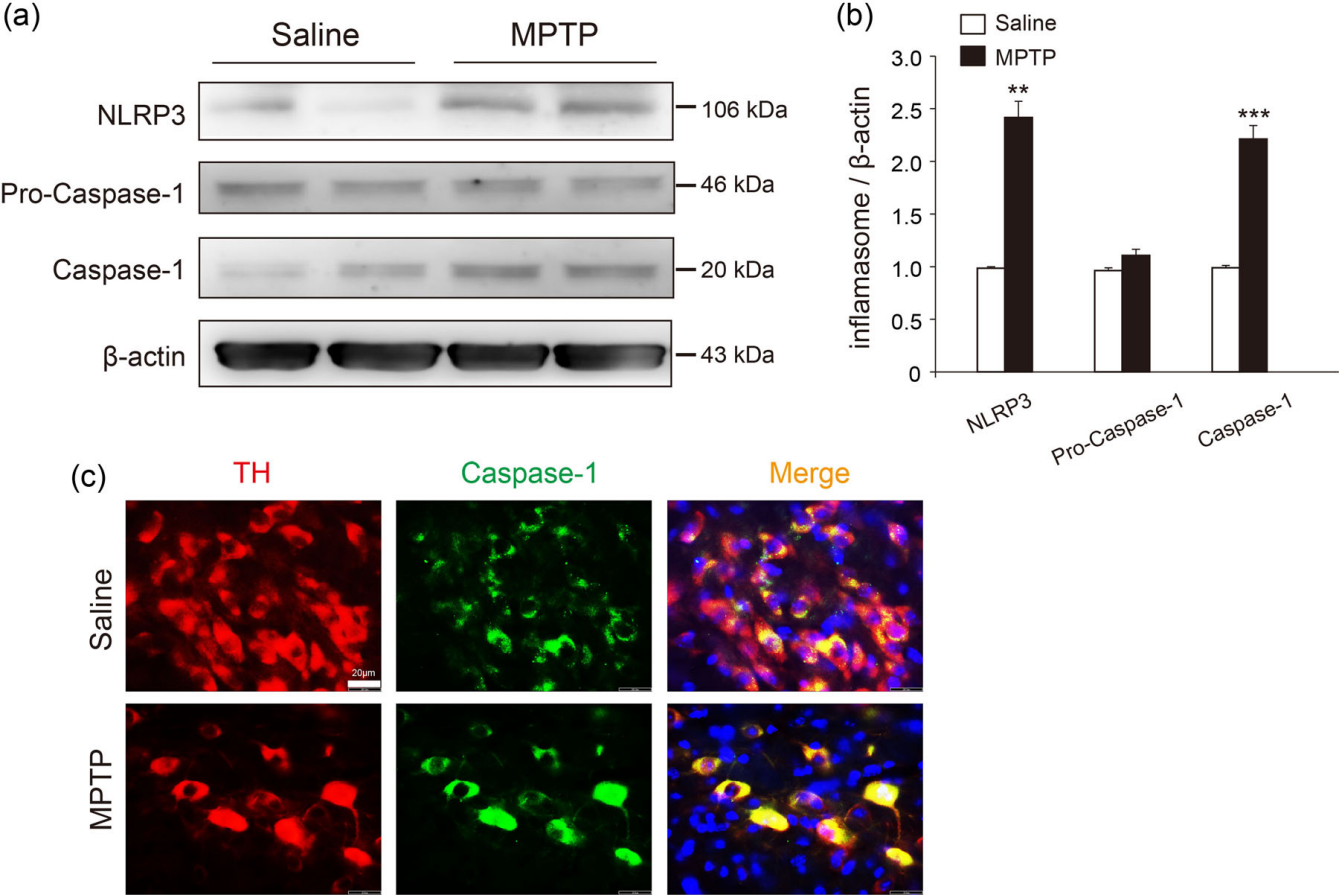
**The NLRP3 inflammasome was activated in acute MPTP mouse model**. (a) Representative blots of NLRP3, pro‐Caspase‐1 and Caspase‐1 expression in the midbrain. (b) Statistical analysis of NLRP3, pro‐Caspase‐1 and Caspase‐1 expression. (c) Double immunofluorescence of TH (red) and Caspase‐1 (green), DAPI stains nucleus (blue), scale bar: 20 μm. Data shown as mean ± SEM (*n* = 6). ***p < .001, vs. saline group (Student's *t* tests). All samples were detected repeatedly in three independent experiments

The acute MPTP mouse model was performed to investigate the role of NLRP3 inflammasome in the development of PD. Compared with Saline group, the expression of NLRP3 was significantly increased 2.3‐fold in the MPTP‐administrated group (Figure 1a). Caspase‐1 is the core component in the assembly of NLRP3 inflammasome and was examined in the midbrain of MPTP‐treated mice. As shown in Figure 1a and b, the expression of Caspase‐1 in the MPTP group was increased extensively without alteration of pro‐Caspase‐1 relative to Saline group, indicating substantial assembly of NLRP3 inflammasome. More importantly, Caspase‐1 was merged with DA neurons reflected by TH and Caspase‐1 immunofluorescence of the SNc sections (Figure 1c). These data suggest that activation of the NLRP3 inflammasome triggers loss of DA neurons and accelerates the pathological process of PD.

### 3.2 Intracerebral stereotactic injection of LV3‐siNlrp3 diminishes the expression of NLRP3 effectively in the SNc region of mice

**FIGURE 2:**
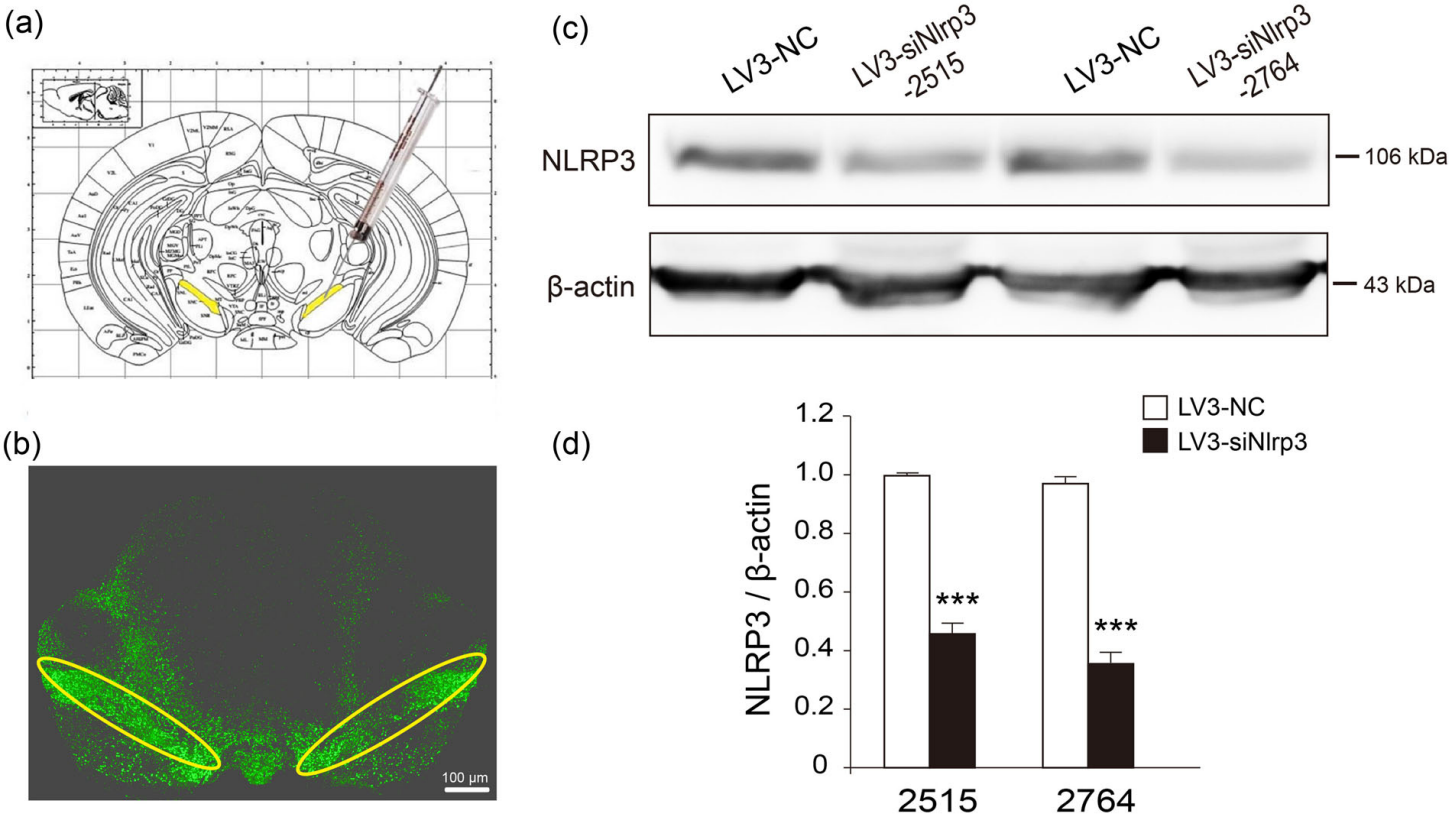
**Intracerebral stereotactic injection with LV3‐siNlrp3 inhibited NLRP3 expression in the SNc of wild‐type mice**. (a) Mouse brain atlas, yellow areas indicate the SNc region (microinjection site). (b) Representative GFP fluorescent image showing the accurate injection of lentivirus into the SNc region of mice, scale bar: 100 μm. (c) Representative blots of NLRP3 expression in the SNc. (d) Statistical analysis of NLRP3 expression. Data shown as mean ± SEM (*n* = 6). ***p < .001, vs. saline group (Student's *t* tests). All samples were detected repeatedly in three independent experiments

Lentivirus‐coated NLRP3 interfering RNA was constructed to determine the effect of the SNc NLRP3 inflammasome activation on PD pathogenesis. According to the brain atlas (Figure 2a), GFP‐labeled control (LV3‐NC), LV3‐siNlrp3‐2515, and LV3‐siNlrp3‐2764 were injected into the SNc of mice, respectively. As shown in Figure 2b, extensive GFP expression was observed in the SNc region with little leakage of lentivirus to surrounding areas, suggesting an accurate delivery of lentivirus to the SNc region of mice (Figure 2b). Delivery of both LV3‐siNlrp3‐2515 and LV3‐siNlrp3‐2764 dramatically reduced the expression of NLRP3 in the SNc (Figure 2c) with a higher efficiency observed in the LV3‐siNlrp3‐2764‐infected mice, showing an inhibition rate of 62% compared with LV3‐NC (Figure 2d). Therefore, LV3‐siNlrp3‐2764 (referred to LV3‐siNlrp3 in the following) was selected as the main lentivirus to inhibit NLRP3 expression in the following experiments.

### 3.3 Knockdown of NLRP3 in the SNc region ameliorates behavioral disorders in MPTP‐treated mice

**FIGURE 3:**
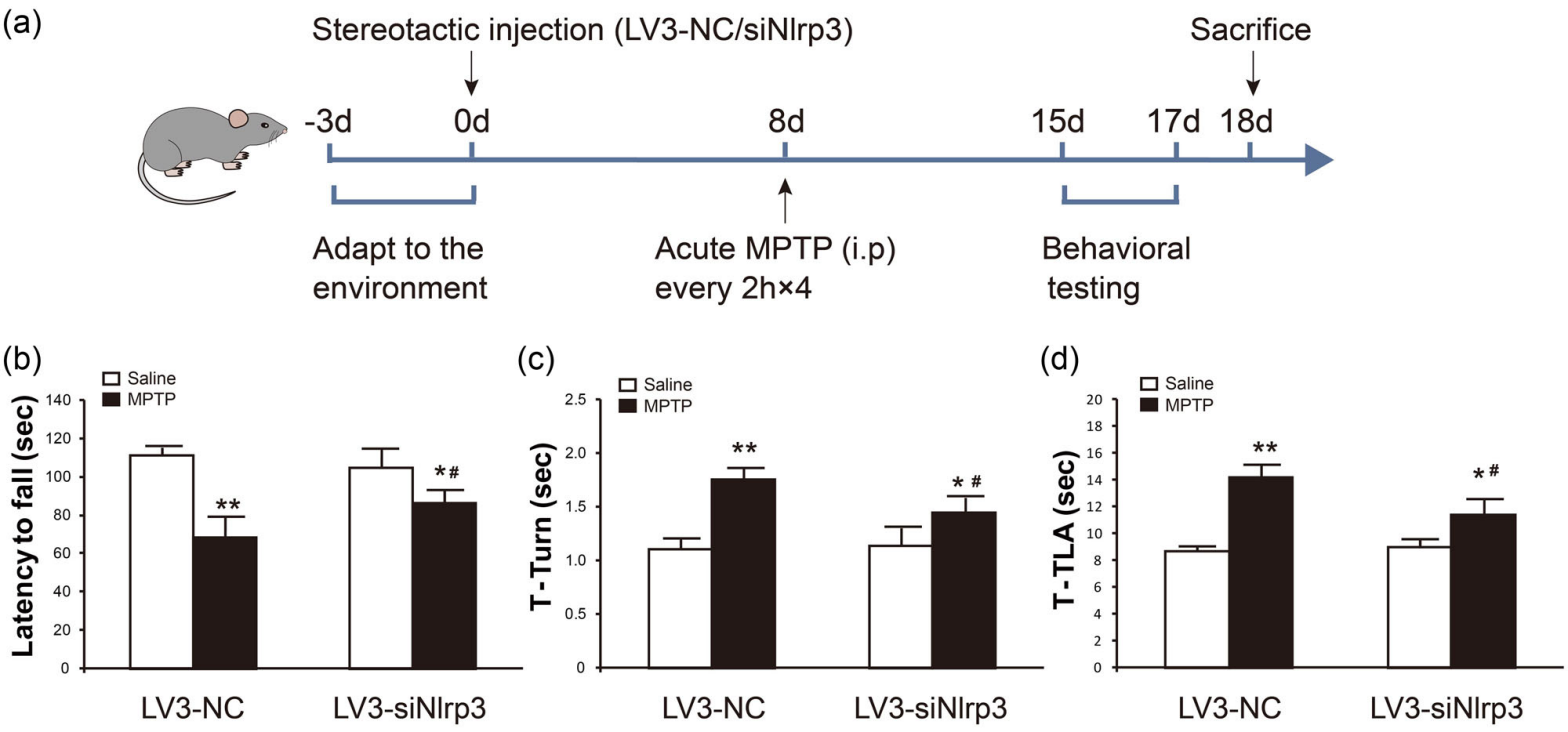
**LV3‐siNlrp3 alleviated MPTP‐induced dyskinesia**. (a) Illustration of experimental design. (b) Mice were trained on the rod for 3 days, and the rotarod test was conducted on the fourth day. The time stayed on the rod was recorded (in seconds). The time of turning around (T‐turn) (c) and descending the pole (T‐TLA) (d) in the pole test. Data are presented as mean ± SEM (*n* = 8), two‐way ANOVA, *p < .05, **p < .01 versus saline groups in corresponding to the treatment of different viruses, ^#^ *p* < .05 versus corresponding LV3‐NC groups

Knockdown of NLPR3 by lentivirus injection into the SNc region was performed prior to MPTP administration followed by behavioral tests (Figure 3a). Rotarod test and pole test are widely used to evaluate motor coordination in the context of PD models. In this study, no difference of motor coordination in baseline was observed between the LV3‐NC and LV3‐siNlrp3 groups (Figure 3b‐d). After acute administration of MPTP, mice displayed significant impaired motor behavior, but LV3‐siNlrp3 administration improved motor symptoms (Figure 3b‐d). MPTP injection caused a prominent decrease in the latencies to fall off the rod (Figure 3b), meanwhile increased the time of turning around and climbing down in the pole test (Figure 3c and d). However, LV3‐siNlrp3 administration significantly improved the performance of MPTP‐treated mice in the rotarod test (Figure 3b) and pole test (Figure 3c and d). These results suggest that inhibition of NLRP3 expression in the SNc region is a promising strategy to improve MPTP‐induced dyskinesia.

### 3.4 Knockdown of NLRP3 in the SNc region prevents MPTP‐induced degeneration of DA neurons

**FIGURE 4:**
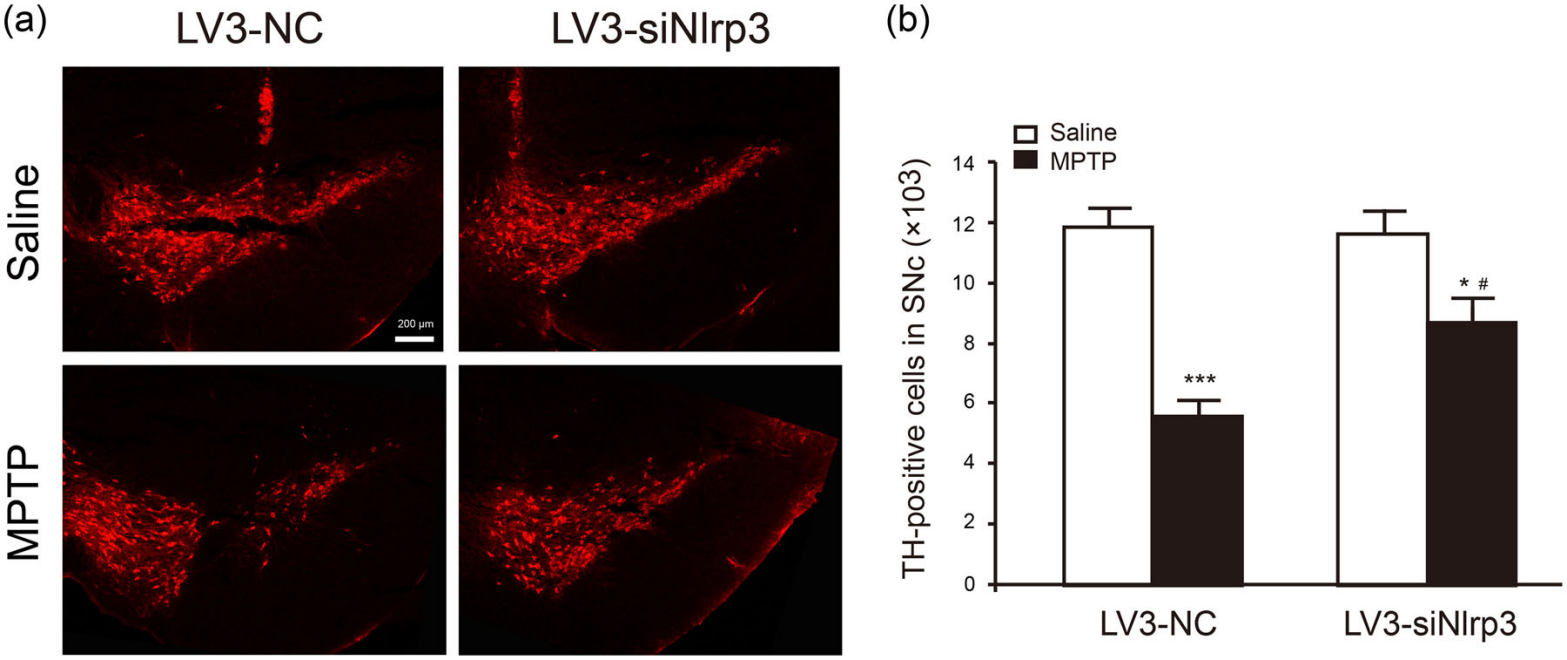
**LV3‐siNlrp3 protected DA neurons against MPTP‐induced damage**. (a) Immunofluorescence images of TH staining (red) in the SNc of each group. Scale bar: 200 μm. (b) Quantitative data for TH‐positive neurons in the SNc. Data are presented as mean ± SEM (*n* = 4), two‐way ANOVA, *p < .05, ***p < .001 versus saline groups in corresponding to the treatment of different viruses; ^#^ *p* < .05 versus corresponding LV3‐NC groups

Progressive loss of DA neurons is the main pathological feature of PD (Bloem et al., 2021). We used immunofluorescence staining to assess the amount of DA neurons after MPTP and LV3‐siNlrp3 treatment. As exhibited in Figure 4a, the density of TH‐positive neurons in the SNc was not affected in LV3‐NC or LV3‐siNlrp3 saline group. After MPTP treatment, the number of TH neurons was reduced by 53% in LV3 NC group, while LV3‐siNlrp3 rescued 54% TH neurons compared to LV3‐NC MPTP group (Figure 4b). These results indicate that NLRP3 knockdown in central SNc provides neuroprotection against PD pathology.

### 3.5 Knockdown of NLRP3 in the SNc region inhibits MPTP‐induced microglia proliferation

**FIGURE 5:**
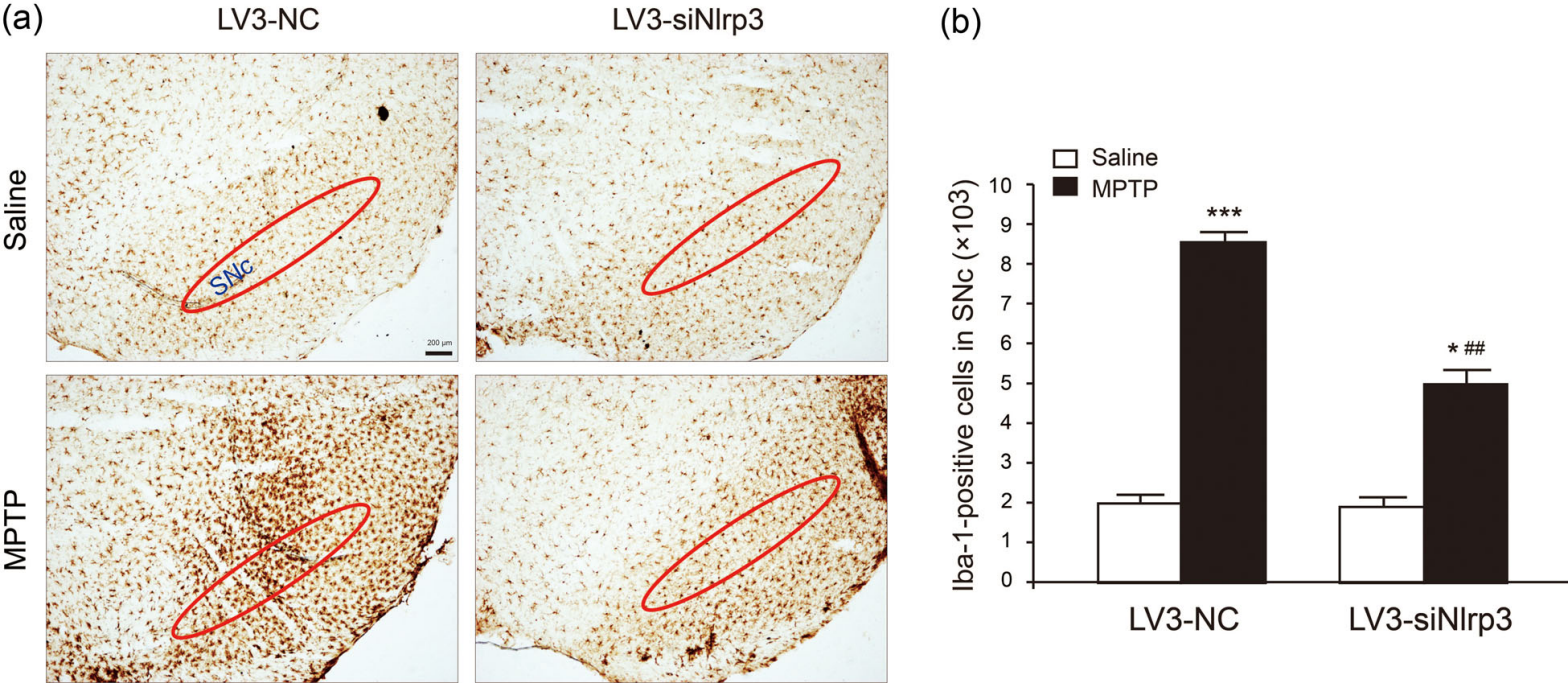
**LV3‐siNlrp3 inhibited MPTP‐induced microgliosis**. (a) Immunohistochemical staining for Iba‐1‐positive microglia (marked in the red oval) in the SNc. Scale bar: 200 μm. (b) Quantitative data for Iba‐1‐positive cells in the SNc. Data are presented as mean ± SEM (*n* = 4), two‐way ANOVA, *p < .05, ***p < .001 versus saline groups in corresponding to the treatment of different viruses; ^#^ *p* < .05 versus corresponding LV3‐NC groups

Our previous research has demonstrated the correlation between NLRP3 inflammasome activation and neuroinflammation in PD progression (Qiao et al., 2018; Zhou et al., 2016). Recently, growing studies have clarified that chronic activation of microglia causes an NLRP3 inflammasome‐mediated release of potent proinflammatory cytokines, which are contributors to neurodegeneration (Haque et al., 2020). In this study, we evaluated the effect of LV3‐siNlrp3 on MPTP‐induced proliferation of microglia by immunohistochemical staining. Obviously, MPTP treatment increased the number of microglia in the SNc (Figure 5a). Iba‐1‐positive cells were increased by 325% in LV3‐NC group, whereas only increased by 163% in LV3‐siNlrp3 group (Figure 5b). Remarkably, the proliferation of microglia induced by MPTP was suppressed in the LV3‐siNlrp3 group. These findings reveal that the neuroprotective effect afforded by NLRP3 knockdown is attributed to the inhibition of microglial proliferation.

### 3.6 Knockdown of microglial NLRP3 protects DA neurons against MPP^+^‐induced injury

**FIGURE 6:**
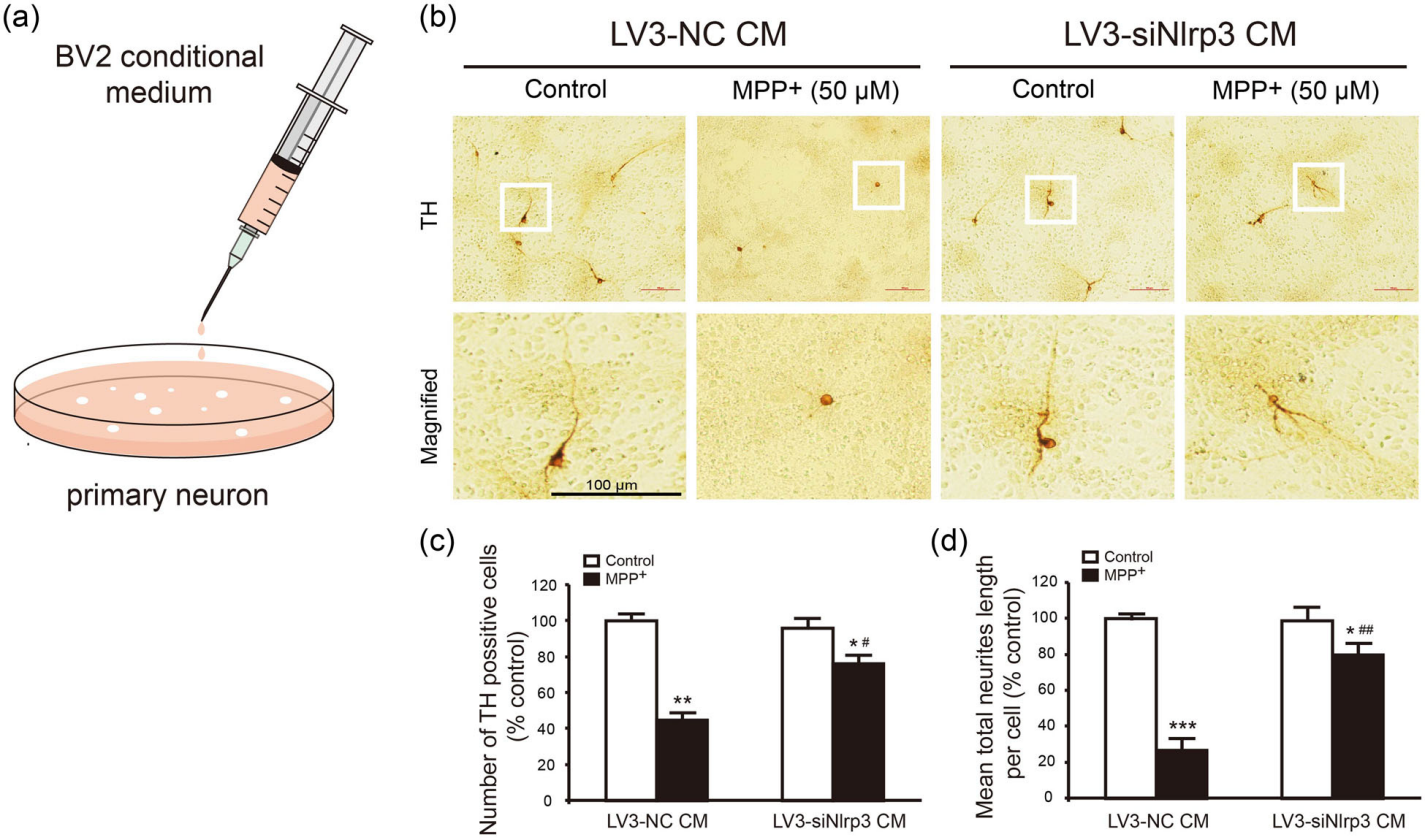
**LV3‐siNlrp3 attenuated neuronal injury via inhibiting NLRP3 expression of BV2 cells in MPP^+^ stimulation**. (a) Diagram of stimulated primary neurons from BV2 supernatant. (b) After treatment with MPP^+^ or lentivirus in BV2 cells, then collected supernatant as microglial conditioned medium (CM) to stimulate primary neurons for 6 h, TH immunohistochemistry detected the DA neurons. The images showed TH‐positive cells. Scale bar: 100 μm. Quantitative analysis of the number of TH‐positive cells (c) and neurite length (d) from four independent experiments. Data are presented as mean ± SEM, two‐way ANOVA, *p < .05, **p < .01, ***p < .001 versus control groups in corresponding to the treatment of different viruses; ^#^ *p* < .05 versus corresponding LV3‐NC groups

Previous studies have shown that neuroinflammation occurs at the early stage of PD and activated microglia release a large number of proinflammatory cytokines, which can aggravate neurodegeneration (Lee et al., 2019; Zhou et al., 2016). To elucidate the direct effect of microglial NLRP3 downregulation on the survival of DA neurons, we used lentivirus to inhibit NLRP3 expression within BV2 cells and collected the conditional medium (CM) to culture primary mesencephalic neurons (Figure 6a). In BV2 cells, with the increase of lentivirus titer, more GFP labeled‐LV3‐NC was expressed (Figure S1), and LV3‐NC or LV3‐siNlrp3 at 10^7^TU/ml was used to infect BV2 cells in the subsequent experiments. The expression of NLRP3 was downregulated by 52% in LV3‐siNlrp3‐infected BV2 cells (Figure S2). After MPP^+^ and lentivirus treatment, the supernatant of BV2 cells was collected as conditional medium (CM) for primary mesencephalic neurons to evaluate the effects of microglia activation on DA neurons survival. As shown in Figure 6b, both LV3‐NC‐CM and LV3‐siNlrp3‐CM had no significant effect on the number and neurite length of TH neurons in control group. Compared with respective control group, MPP^+^ dramatically reduced the number of TH neurons and shortened the length of neurites (Figure 6b). Nonetheless, neurons treated with LV3‐siNlrp3‐CM but not LV3‐NC‐CM was resistant to MPP^+^‐induced injury (Figure 6c and d). These data strengthen the detrimental role of microglial NLRP3 in PD progression and provide a potential disease‐modifying therapy for PD.

## 4 DISCUSSION

Although the pathogenesis of PD remains unclear, increasing evidence has illustrated that neuroinflammation is a key player in PD (Badanjak et al., 2021). Substantial proinflammatory cytokines are found in PD patients (Zhou et al., 2016), leading to numerous studies into the development of anti‐inflammatory drugs for PD therapy (Shah et al., 2018; Walsh et al., 2015). However, no available drugs are developed successfully based on anti‐inflammation hypothesis for PD therapy. To this end, beyond addressing our knowledge gaps concerning the intricate role of neuroinflammation in PD pathogenesis, the present study also aimed to provide a potential disease‐modifying strategy by targeting microglial NLRP3 in the SNc to treat PD.

Microglia is the first line of defense for neuroimmunity in the brain, and the activation of microglia releases abundant inflammatory factors, which aggravated the development of PD (Ho, 2019). In this study, we found that MPTP‐treated mice exhibited proliferation and hyperactivation of microglia in the SNc region, indicating a detrimental role of microgliosis in PD progression. As we known, neuronal pyroptosis and neuroinflammation mediated by NLRP3 inflammasome play an important role in neurodegenerative diseases, which are important regulators of microglial neuroinflammation (Ahmed et al., 2021; Gordon et al., 2018; Haque et al., 2020; Li et al., 2021; Ramesh et al., 2013), we next sought to explore the effects of microglial NLRP3 on DA neurodegeneration. LV3‐siNlrp3 was constructed and injected into the SNc region of MPTP‐treated mice. Intriguingly, LV3‐siNlrp3 treatment significantly inhibited microgliosis and attenuated PD pathology.

Unlike previous studies, which mostly demonstrated the role of NLRP3 in PD pathogenesis by using NLRP3 knockout mice or inhibitors (Guo et al., 2017; Lee et al., 2019; Ou et al., 2021), our study clarified region‐specific effects of NLRP3 on PD pathology that provides straightforward evidence for the association between NLRP3 and PD, and demonstrated the feasibility of disease‐modifying therapy in PD mouse model.

The NLRP3 inflammasome is an oligomeric complex consisting of NLRP3, Caspase‐1 and ASC, responsible for microglia activation‐mediated neuroinflammation in the brain (Holbrook et al., 2021). To uncover the role of microglia‐neuron communication in PD pathogenesis, an indirect coculture system was performed in our study to investigate the effect of microglial NLRP3 on DA neurons survival in the context of MPP^+^ stimulation. Consistent with our in vivo results, conditional medium collected from LV3‐siNlrp3‐treated microglia increased the resistance of DA neurons to MPP^+^‐induced injury. These findings directly depict microglial NLRP3‐induced inflammation as the murderer of DA neurons and that microglial NLRP3 knockdown is sufficient to block destructive influence on DA neurons survival from microglia‐neuron communication.

**FIGURE 7:**
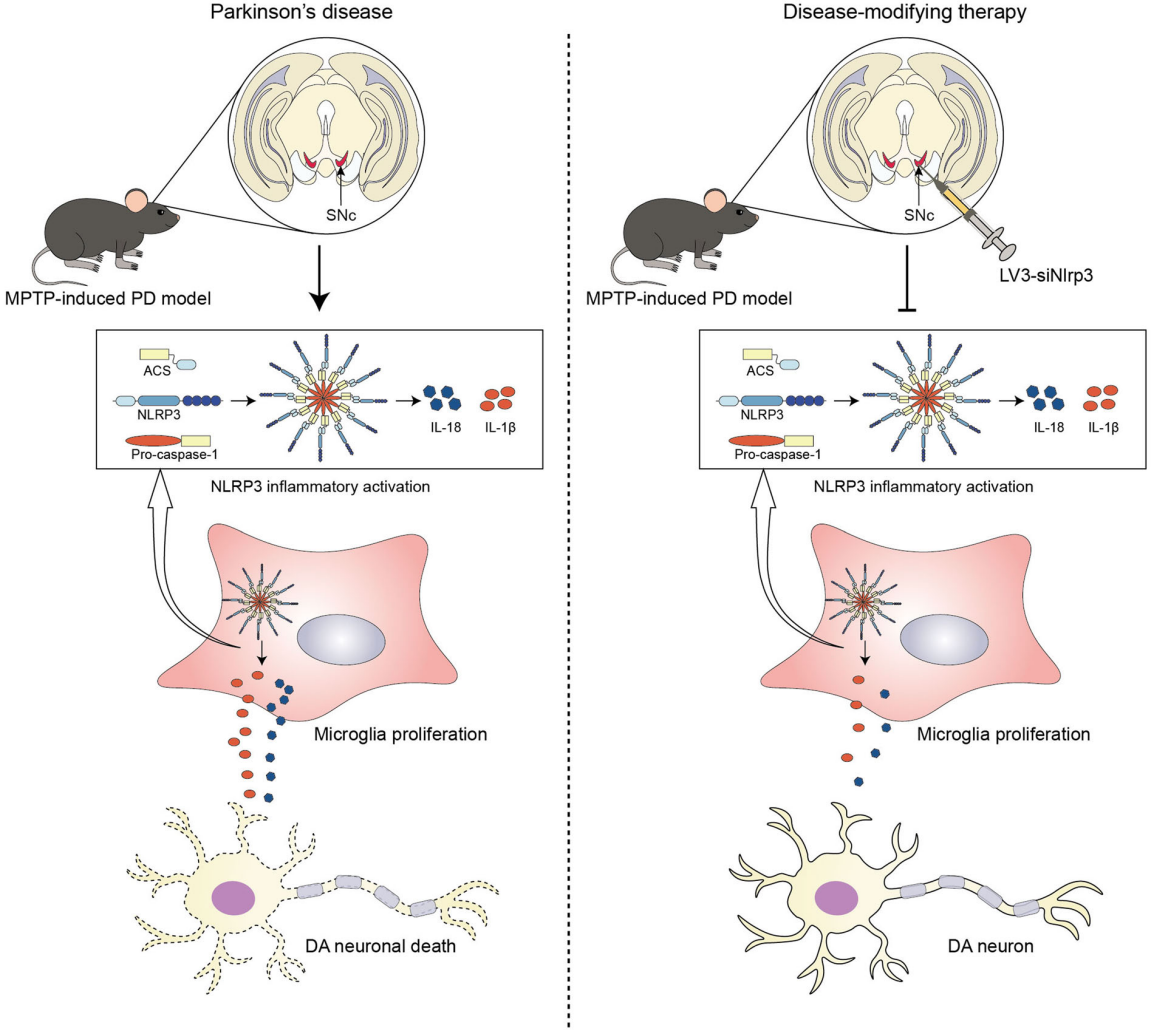
**Schematic model demonstrated that NLRP3 downregulation in the SNc restricted microgliosis and protected dopaminergic neurons in Parkinson's disease mouse model**. LV‐siNlrp3 alleviated microglia‐mediated neuroinflammation and rescued DA neurons in PD model mice through disease‐modifying therapy

Collectively, our study elucidates that knockdown of NLRP3 in the SNc region suppresses microglia proliferation and hyperactivation through inhibition of NLRP3 inflammasome activation, exerting protective effects on DA neurons (Figure 7). However, posttreatment of LV3‐siNlrp3 is warranted in future research to provide a comprehensive view of microglial NLPR3 as a valuable therapeutic target for PD. Nevertheless, this work not only fills the gap in our understanding of the role of NLRP3 in microglia‐neuron communication, but also provides a promising disease‐modifying target for PD therapy.

## FUNDING

The work reported herein was supported by the grants from the National Natural Science Foundation of China (No. 81803505, 82003722), Medical Research Project of Jiangsu Provincial Health Commission (M2022071), Health Industry clinical research special project of Shanghai Municipal Health Commission (Grant No.20204Y0462), Jiangsu Research Hospital Association for Precision Medication (JY202134), and Jinshan Yingcai “169 ” project of Zhenjiang.

## CONFLICT OF INTEREST

The authors declare no conflict of interest.

### PEER REVIEW

The peer review history for this article is available at https://publons.com/publon/10.1002/brb3.2784

## Supporting information


**Figure S1**. The titer of lentivirus transfection was explored in BV2 cells. GFP intensity showing the transfection efficiency of lentivirus, scale bar: 100 μm.

**Figure S2. Knockdown efficiency of LV‐siNlrp3 was detected in BV2 cells**. After transfection for 24 h, NLRP3 expression was tested by western blot. (a) Representative blots of NLRP3 in BV2 cells. (b) Statistical analysis of NLRP3 expression. Data are expressed as mean ± SEM, ***p < .001, *vs*. LV3‐NC group (Student's *t* tests). All samples were detected repeatedly in three independent experiments.

## Data Availability

The data used to support the findings of this study are available from the corresponding author upon request.
