# Supplementary figures and images for: Targeting microglial NLRP3 in the SNc region as a promising disease‐modifying therapy for Parkinson's disease
Source: Brain Behav. 2022 Oct 5;12(11):e2784. doi: 10.1002/brb3.2784 (PMC9660492; doi:10.1002/brb3.2784)

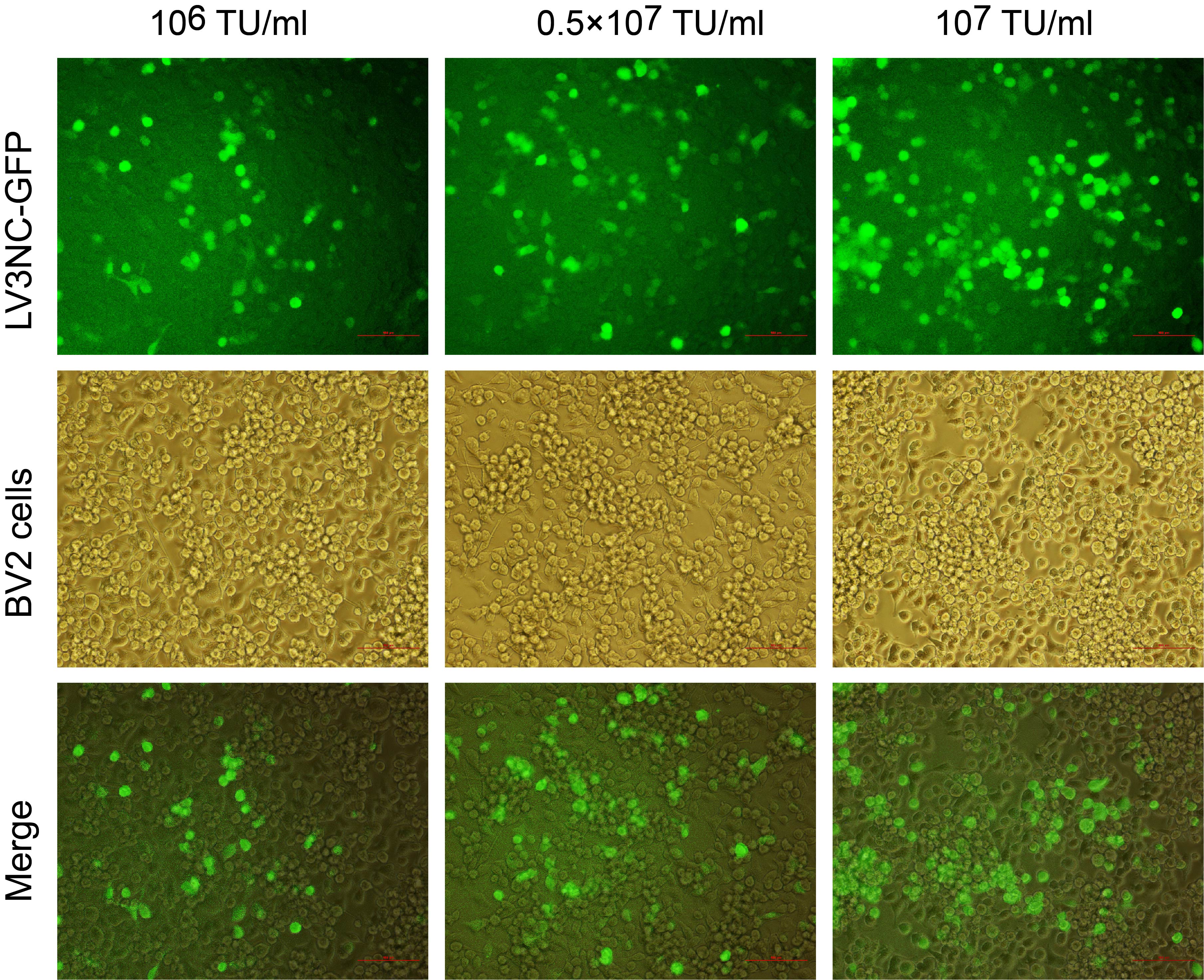

Supplement: Supplementary file 1 — Figure S1. The titer of lentivirus transfection was explored in BV2 cells. GFP intensity showing the transfection efficiency of lentivirus, scale bar: 100 μm. [file BRB3-12-e2784-s001.jpg]

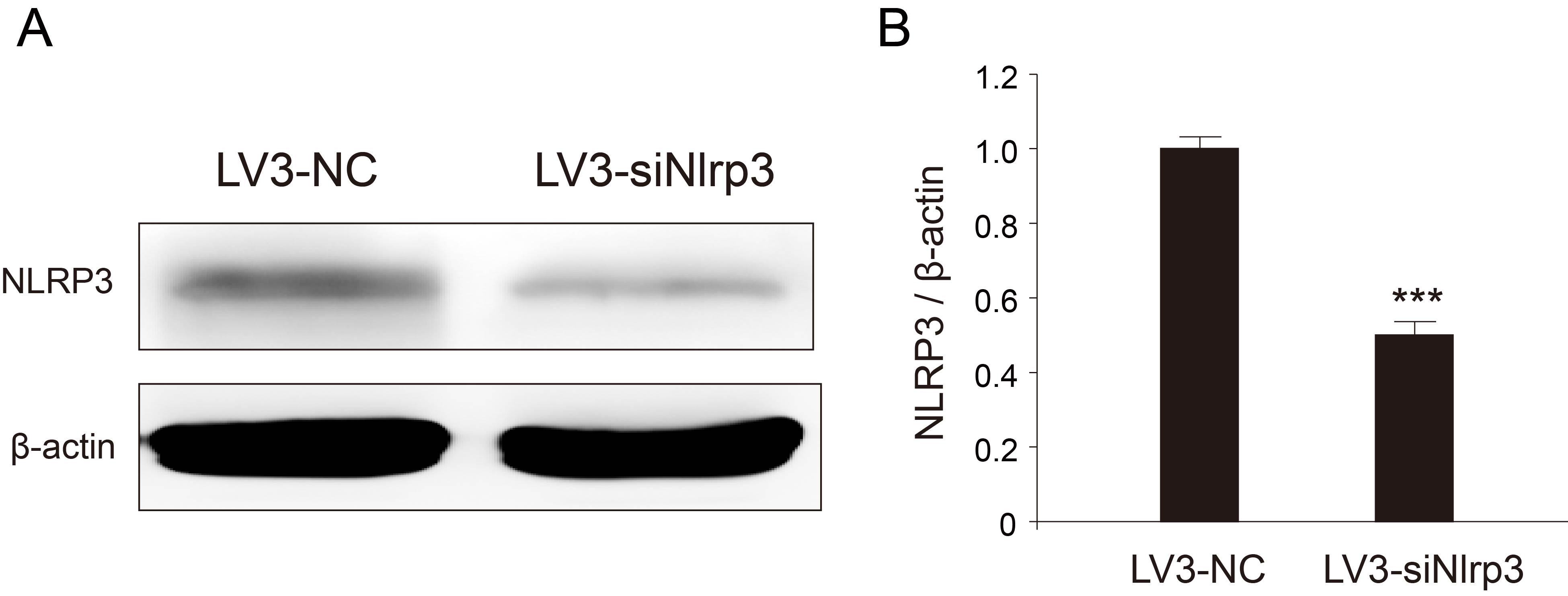

Supplement: Supplementary file 2 — Figure S2. Knockdown efficiency of LV‐siNlrp3 was detected in BV2 cells. After transfection for 24 h, NLRP3 expression was tested by western blot. (a) Representative blots of NLRP3 in BV2 cells. (b) Statistical analysis of NLRP3 expression. Data are expressed as mean ± SEM, ***p < .001, vs. LV3‐NC group (Student's t tests). All samples were detected repeatedly in three independent experiments. [file BRB3-12-e2784-s002.jpg]
